# Evaluation of UNI-VAC serum separator, ethylenediaminetetraacetic acid, sodium citrate, and sodium fluoride tubes: A comparison with Becton, Dickinson and Company Vacutainer tubes

**DOI:** 10.1371/journal.pone.0349489

**Published:** 2026-10-05

**Authors:** Kuenyoul Park, Joonsang Yu, Wonho Choe, Soo Jin Yoo

**Affiliations:** 1 Department of Laboratory Medicine, Hanyang University Seoul Hospital, Hanyang University College of Medicine, Seoul, Republic of Korea; 2 Department of Laboratory Medicine, Sanggye Paik Hospital, Inje University College of Medicine, Seoul, Republic of Korea; 3 Department of Laboratory Medicine, Uijeongbu Eulji Medical Center, Eulji University, Uijeongbu, Republic of Korea; Children’s Hospital of Orange County, UNITED STATES OF AMERICA

## Abstract

Errors in the preanalytical phase are influenced by several factors, including blood collection tubes. Labex, Inc. has developed UNI-VAC tubes using a separation gel formulated with fatty acid acrylamide to enhance stability. We aimed to compare the performance of the UNI-VAC tubes with Becton, Dickinson and Company Vacutainer tubes for 53 analytes. Forty adult outpatients were prospectively enrolled in the comparison study, and an additional 20 participants were included to evaluate lot-to-lot variability. Four UNI-VAC tube types (serum separator, ethylenediaminetetraacetic acid [EDTA], sodium citrate, and sodium fluoride) were compared with their Vacutainer counterparts. Comparative performance was evaluated using Passing–Bablok regression and bias criteria from the European Federation of Clinical Chemistry and Laboratory Medicine Biological Variation Database and Westgard specifications. Stability was assessed for EDTA tubes at 24 h and sodium fluoride tubes at 24–72 h (4°C storage). The lot-to-lot variability of the EDTA tubes was examined across the three UNI-VAC and two Vacutainer lots. Fifty (94%) analytes met the predefined acceptability criteria. Lactate dehydrogenase (−4.3%), mean corpuscular volume (1.5%), and mean corpuscular hemoglobin concentration (−1.0%) marginally exceeded these limits, although the differences were small and unlikely to be clinically significant. No visible gel instability or other visible tube-related issues were observed. EDTA tubes demonstrated stability after 24 h, although the basophil percentages and platelet indices showed reduced consistency. Glucose values were stable for up to 48 h but declined at 72 h in both tubes. The lot-to-lot variability of the UNI-VAC EDTA tubes was comparable to that of the Vacutainer tubes. UNI-VAC tubes show a clinical performance equivalent to that of Vacutainer tubes for most analytes, with acceptable stability. These findings indicate that UNI-VAC tubes represent a reliable alternative for clinical laboratory use, with further validation in larger patient cohorts.

## Introduction

Errors occurring in the preanalytical phase are significantly influenced by the performance of blood collection tubes, making their quality a critical determinant of reliable laboratory testing [1,2]. Potential problems include incorrect additives or volumes, defective separator gels, and interactions with tube materials [3–5]. Although separator gels in plasma or serum separation tubes are generally designed to be chemically inert, they can alter analyte concentrations [6]. To address these issues, Labex, Inc. (Seoul, Korea) recently introduced the UNI-VAC tube, which uses a separation gel formulated with fatty acid acrylamide. According to the manufacturer, the amide moieties of the fatty acid acrylamide participate in hydrogen bonding to form hydrogen bond chains, which are claimed to promote a more effective three-dimensional network structure, thereby enhancing the physical stability of the gel [7]. This improved structural integrity is suggested to reduce the likelihood of gel particle formation, commonly referred to as oil droplets, even under conditions of temperature fluctuation or prolonged storage, potentially ensuring a more stable separation performance after centrifugation. To our knowledge, its analytical performance has not previously been evaluated independently. The present study therefore provides the first independent assessment of whether this gel maintains analytical comparability with the established blood collection tube system under routine clinical-laboratory conditions. Because blood collection tubes can be a source of systematic or random error in analyte measurement, their evaluation against an established reference system before clinical adoption is essential to ensure accurate and reliable patient results [3–6].

This study aimed to evaluate the applicability of UNI-VAC tubes in clinical laboratories, including serum separator with gel, ethylenediaminetetraacetic acid (EDTA), sodium citrate, and sodium fluoride (for glucose) tubes. Their performance was compared with that of Becton, Dickinson and Company (BD) Vacutainer tubes manufactured by BD (Franklin Lakes, NJ, USA) through paired sample collection and immediate parallel testing. The UNI-VAC tubes were assessed for their physical characteristics during preanalytical procedures, such as blood collection and centrifugation, and for their analytical performance across 53 laboratory tests routinely performed at our institution, spanning clinical chemistry, diagnostic immunology, hematology, and coagulation. Furthermore, we aimed to evaluate the stability of the EDTA tubes and conducted a preliminary assessment of lot-to-lot variability.

## Materials and methods

### Study population

This study prospectively enrolled 60 adult participants following approval from the Institutional Review Board (IRB). According to the CLSI EP09-A3 guideline [8], at least 40 participants are recommended for method comparison studies, with an additional 20 participants enrolled for the evaluation of lot-to-lot variability. The participants were adults (≥19 years) who visited the hospital’s outpatient phlebotomy unit and provided written informed consent prior to enrollment. The exclusion criteria were as follows: (i) a history of bleeding disorders, severe anemia, or cytopenia; (ii) prior adverse reactions to venipuncture (e.g., hematoma, vasovagal syncope); (iii) body weight <50 kg; (iv) pregnancy; and (v) clinical requirement for ≥25 mL of blood sampling for medical purposes, which in combination with the study sampling would result in a total sampling volume >40 mL. These exclusions were applied to protect potentially vulnerable participants from the additional research-only venipuncture required by the study protocol. The demographic characteristics of the participants were not collected, as this study focused solely on preanalytical tube performance.

### Blood collection and handling

Blood was collected from each participant by venipuncture into four tubes (Labex, Inc., Seoul, Korea): a serum separation tube (branded as serum with gel, 5 mL), EDTA tube (3 mL), sodium citrate tube (2.78 mL), and sodium fluoride tube (2 mL), totaling approximately 25.4 mL per participant. One tube of each type was used per participant, with a single lot used for each tube type per manufacturer (one lot per manufacturer), and the corresponding BD Vacutainer tubes of equivalent volumes served as comparators. To minimize any potential effect of draw order on the comparative results, the order in which the two manufacturers’ tubes were collected was systematically alternated across participants, such that BD Vacutainer and UNI-VAC tubes were drawn first in an alternating sequence. All venipunctures were performed using a single type of needle and needle holder (BD) to eliminate device-related variability. Blood was collected in the following order: citrate, serum separator, EDTA, and sodium fluoride tubes. Visual inspection was conducted immediately after centrifugation to assess hemolysis, fibrin formation, and gel abnormalities and to confirm adequate phase separation. Hemolysis was evaluated qualitatively by visual inspection only.

For lot-to-lot variability testing, blood samples (15 mL in total) were collected from the other 20 participants, with 3 mL allocated to each of the following five EDTA tubes: two BD Vacutainer EDTA tubes (from two different lots) and three UNI-VAC EDTA tubes (from three different lots).

For the primary method-comparison analysis, all samples were processed without delay at room temperature to isolate tube-related effects. Separately, for the stability sub-study, the designated EDTA and sodium fluoride tubes were first analyzed at baseline under these same immediate-processing conditions; the same tubes were then stored at 4 °C and reanalyzed at the predefined time points (EDTA at baseline and 24 h; sodium fluoride at baseline, 24, 48, and 72 h) to assess time-dependent analyte stability. The serum separation tubes were allowed to clot for 30 min before centrifugation at 2,000 × g for 10 min. Sodium fluoride tubes were centrifuged within 30 min of collection at 2,000 × g for 10 min. The EDTA tubes were mixed by gentle inversion and analyzed within 2 h of collection. The sodium citrate tubes were centrifuged at 2,000 × g for 10 min. Paired samples from the UNI-VAC and BD Vacutainer tubes and samples from different lots were tested in the same analytical run to ensure comparability and minimize batch-to-batch variability. Serum separation and sodium fluoride tubes were analyzed using an AU5800 automated clinical chemistry analyzer (Beckman Coulter, Brea, CA, USA). Hematological tests were performed using XN-20 (Sysmex, Kobe, Japan). Prothrombin time and activated partial thromboplastin time were measured using STA R Max3 (Diagnostica Stago, Asnières-sur-Seine, France). Hemoglobin A1c levels were measured using D-100 (Bio-Rad, Hercules, CA, USA). Thyroid hormones (thyroid-stimulating hormone [TSH], free thyroxine, and total triiodothyronine) were analyzed using dedicated *Elecsys TSH, Elecsys FT4 III*, *and Elecsys T3* reagents on a Cobas 8000 analyzer (Roche Diagnostics GmbH, Mannheim, Germany). The study laboratory was accredited by the Laboratory Medicine Foundation of Korea and participated in the Korean Association of External Quality Assessment Service program.

### Comparison study

The comparability between BD Vacutainer and UNI-VAC tubes was assessed using 53 routine laboratory tests (Table 1), including clinical chemistry, diagnostic immunology, hematology, coagulation, and glucose assays. Statistical evaluation included calculation of the mean percentage difference, Passing–Bablok regression, and paired t-tests. Passing–Bablok regression was considered acceptable when the 95% confidence interval (CI) of the slope included 1.0 and that of the intercept included 0, indicating the absence of proportional and constant biases. Paired t-tests were used to determine statistically significant differences between the two blood collection tubes, with P < 0.05 considered statistically significant. Acceptability criteria were based on the desirable bias limits obtained from the European Federation of Clinical Chemistry and Laboratory Medicine **(**EFLM) Biological Variation Database (https://biologicalvariation.eu/; accessed May 19, 2025). For direct bilirubin and hematological parameters, the Westgard Desirable Biological Variation specifications (https://westgard.com/clia-a-quality/quality-requirements/biodatabase1.html; accessed May 19, 2025) were used. For platelet distribution width (PDW) and plateletcrit (PCT), where desirable bias values were unavailable, desirable imprecision values were used. For the unsaturated iron-binding capacity, one-quarter of the CLIA 2025 (https://westgard.com/clia-a-quality/quality-requirements/2024-clia-requirements.html; accessed May 19, 2025), the allowable total error (5%) was used. Bland–Altman analysis, including graphical plots, was additionally performed to visualize mean bias and 95% limits of agreement between the two tubes.

**Table 1:** Blood collection tubes evaluated, corresponding analytes, and manufacturer lot numbers.

Tube type	Measured analytes	Lot number (Labex UNI-VAC)	Lot number (BD Vacutainer)
5 mL serum separation tube^*^	Total protein, albumin, glucose, total bilirubin, direct bilirubin, blood urea nitrogen, creatinine, aspartate aminotransferase, alanine aminotransferase, lactate dehydrogenase, gamma-glutamyl transferase, alkaline phosphatase, creatine kinase, sodium, potassium, chloride, cholesterol, C-reactive protein, uric acid, calcium, inorganic phosphorus, iron, unsaturated iron-binding capacity, magnesium, triglyceride, high-density lipoprotein cholesterol, low-density lipoprotein cholesterol, amylase, lipase, triiodothyronine, thyroid-stimulating hormone, free thyroxine	1EC141	4204755
3 mL K_2_ EDTA	White blood cell count, red blood cell count, hemoglobin, hematocrit, mean corpuscular volume, mean corpuscular hemoglobin, mean corpuscular hemoglobin concentration, red cell distribution width, neutrophil percentage, lymphocyte percentage, monocyte percentage, eosinophil percentage, basophil percentage, platelet count, mean platelet volume, platelet distribution width, plateletcrit, hemoglobin A1c	2EC141, 2EB142, 2EA202	4222910, 4257248
2.78 mL sodium citrate	Prothrombin time, activated partial thromboplastin time	2EC191	4281719
2 mL sodium fluoride	Glucose	2EB201	4045107

* The serum separation tube manufactured by Labex was marketed under the brand name “UNI-VAC Serum with gel.”

### Stability assessment

The stability was assessed using EDTA and sodium fluoride tubes. EDTA samples were analyzed at baseline and at 24 ± 2 h. Sodium fluoride samples were analyzed at baseline and at 24 ± 2 h, 48 ± 2 h, and 72 ± 2 h. During the study period, the samples were stored at 4°C to preserve analyte stability. Stability was evaluated by comparing the mean percentage difference against the allowable bias criteria.

### Lot-to-lot variability

The lot-to-lot variability of the EDTA tubes was evaluated using three UNI-VAC lots and two BD Vacutainer lots. The standard deviation (A) of the lot results was calculated using the modified CLSI GP34-A:2010 approach [9]:


$$\begin{array}{c} A=\sqrt{\frac{\sum_{i=\text{1}}^{N}\sum_{j=\text{1}}^{L}{\left(y_{ij}-{\overset{―}{y}}_{i.}\right)}^{\text{2}}}{N(L-\text{1})}} \end{array}$$

where A is the standard deviation of the lot results, N is the total number of participants, L is the number of tube lots, $y_{ij}$is the result of tube lot j from participant i, and ${\overset{―}{y}}_{i.}$is the average of all observations for participant i. Lot variability between the two tube manufacturers was compared using F-tests.

### Statistical analyses

All analyses were performed using the R software (version 4.5.0). Data preprocessing and summarization were performed using the dplyr, tidyr, readr, and tibble packages. Visualization was conducted using ggplot2. Passing–Bablok regression was performed using the mcr package. Paired t- and F-tests were conducted using the stats package. A two-tailed P-value <0.05 was considered statistically significant for all tests.

### Ethics approval

This study was conducted in accordance with the principles of the Declaration of Helsinki and approved by the IRB of Sanggye Paik Hospital (IRB No. SPIRB-2025-01-009). Participants were recruited from April 7, 2025, to April 21, 2025. Written informed consent was obtained from all participants before enrollment.

## Results

### Study participants and overall tube integrity

Forty adult outpatients were enrolled for method comparison and stability testing. An additional 20 participants were recruited for lot-to-lot variability assessment. All venipunctures were successfully performed with adequate sample volumes. No specimens were excluded due to clotting, inadequate volume, or other preanalytical issues. No abnormal findings, such as incomplete phase separation or gel detachment, were observed during centrifugation in either BD Vacutainer or UNI-VAC tubes.

### Comparison between UNI-VAC and Becton, Dickinson and Company Vacutainer

Across 53 routine laboratory tests, most analytes showed mean percentage differences within the predefined allowable bias limits. Three analytes exceeded these limits: lactate dehydrogenase (LDH, −4.26%; limit 3.10%), mean corpuscular volume (MCV, 1.45%; limit 1.26%), and mean corpuscular hemoglobin concentration (MCHC, −0.97%; limit 0.40%). For LDH, the difference remained below both the minimum allowable bias from the EFLM Biological Variation Database (4.7%) and the Westgard desirable bias (4.3%). Paired t-tests identified statistically significant differences in 20 analytes, including total protein, glucose, total bilirubin, blood urea nitrogen, creatinine, sodium, uric acid, calcium, inorganic phosphorus, lipid parameters, TSH, and several hematological indices; apart from LDH, MCV, and MCHC noted above, the corresponding mean differences remained within the acceptable bias limits. Passing–Bablok regression analyses revealed that all analytes had 95% CIs for the slope that included 1.0. However, some analytes (glucose, creatinine, total bilirubin, high-density lipoprotein cholesterol, white blood cell count, and glucose in sodium fluoride tubes) had 95% CIs of the intercept that did not include 0. For C-reactive protein (CRP), most values were below the lower limit of quantification (0.3 mg/dL); therefore, a regression analysis was not performed (Table 2). Bland–Altman analysis confirmed small biases between the two tube types, within the allowable-bias limits for all analytes except LDH, MCV, and MCHC (S1 Fig).

**Table 2:** Comparison of 53 analyte measurements in UNI-VAC and BD Vacutainer tubes.

Tube	Item	Unit	Measured range (BD Vacutainer)	Mean % difference	Passing–Bablok slope (95% CI)	Passing–Bablok intercept (95% CI)	Paired t-test P-value	Allowable bias (%)
SST	Total protein	g/dL	6.54–8.57	–0.50%	0.95 (0.90–1.00)	0.32 (–0.05–0.69)	0.0022	1.30
SST	Albumin	g/dL	4.06–5.25	–0.16%	1.00 (0.95–1.06)	–0.01 (–0.27–0.20)	0.2613	1.20
SST	Glucose	mg/dL	69.00–368.00	–1.32%	1.00 (1.00–1.02)	–1.00 (–4.07 to –1.00)	0.0001	2.30
SST	Total bilirubin	mg/dL	0.25–1.20	–1.08%	1.00 (1.00–1.02)	–0.01 (–0.02 to –0.01)	0.0213	8.00
SST	Direct bilirubin	mg/dL	0.04–0.25	2.50%	1.00 (0.98–1.00)	0.00 (0.00–0.00)	0.3611	14.20
SST	Blood urea nitrogen	mg/dL	5.40 ~ 47.40	–1.61%	0.98 (0.96–1.00)	0.09 (–0.17–0.29)	0	6.00
SST	Creatinine	mg/dL	0.46–2.91	–1.14%	1.00 (1.00–1.05)	–0.01 (–0.05 to –0.01)	0.0112	4.20
SST	Aspartate aminotransferase	U/L	11.70–87.10	0.42%	1.01 (0.99–1.04)	–0.18 (–0.76–0.39)	0.3017	5.30
SST	Alanine aminotransferase	U/L	9.40–79.70	0.42%	0.99 (0.97–1.01)	0.20 (–0.17–0.73)	0.3855	9.20
SST	Lactate dehydrogenase	U/L	230.00–547.00	–4.26%	0.99 (0.89–1.10)	–7.75 (–48.25–28.07)	0	3.10
SST	Gamma-glutamyl transferase	U/L	8.70–254.80	0.43%	1.00 (1.00–1.01)	0.05 (–0.24–0.19)	0.1752	11.50
SST	Alkaline phosphatase	U/L	39.00–150.00	0.65%	1.00 (0.99–1.02)	0.00 (–1.03–1.17)	0.055	5.50
SST	Creatine kinase	U/L	37.00–1928.00	–0.76%	1.00 (0.99–1.00)	–0.50 (–0.50–0.77)	0.1193	8.90
SST	Sodium	mmol/L	136.00–143.00	0.20%	1.00 (1.00–1.00)	0.00 (0.00–0.00)	0.0098	0.20
SST	Potassium	mmol/L	3.60–5.20	–0.56%	1.00 (1.00–1.08)	0.00 (–0.39–0.00)	0.2627	1.70
SST	Chloride	mmol/L	99.00–111.00	–0.05%	1.00 (1.00–1.00)	0.00 (0.00–0.00)	0.5703	0.40
SST	Cholesterol	mg/dL	106.10–258.30	0.02%	1.00 (0.98–1.01)	0.58 (–2.37–3.42)	0.9848	4.10
SST	C-reactive protein	mg/dL	–0.03–7.31	4.72%	N/A	N/A	0.2163	23.40
SST	Uric acid	mg/dL	3.00–8.08	–0.58%	0.99 (0.99–1.00)	0.00 (–0.03–0.03)	0	6.00
SST	Calcium	mg/dL	9.30–10.51	0.27%	1.01 (0.93–1.13)	–0.11 (–1.23–0.71)	0.0318	0.70
SST	Inorganic phosphorus	mg/dL	2.58–4.72	–0.80%	0.98 (0.96–1.00)	0.05 (–0.04–0.11)	0.0002	3.30
SST	Iron	μg/dL	20.00–187.00	–0.30%	1.00 (0.99–1.01)	–0.50 (–1.63–0.11)	0.1658	9.60
SST	Unsaturated iron-binding capacity	μg/dL	147.00–397.00	0.31%	0.98 (0.96–1.01)	4.26 (–1.63–9.34)	0.4123	5.00
SST	Magnesium	mg/dL	1.76–2.45	–0.03%	1.00 (0.98–1.07)	0.00 (–0.14–0.05)	0.8516	1.60
SST	Triglyceride	mg/dL	37.00–389.00	0.06%	1.01 (1.00–1.02)	–0.50 (–1.99–0.00)	0.4939	9.90
SST	High-density lipoprotein cholesterol	mg/dL	39.00–84.00	3.37%	1.00 (0.96–1.00)	2.00 (2.00–4.93)	0	5.50
SST	Low-density lipoprotein cholesterol	mg/dL	36.00–164.00	0.75%	1.00 (1.00–1.02)	0.50 (–1.70–0.50)	0.0067	5.60
SST	Amylase	U/L	28.00–145.00	–0.45%	1.00 (1.00–1.00)	0.00 (0.00–0.00)	0.1519	7.50
SST	Lipase	U/L	6.00–66.00	–1.93%	1.00 (1.00–1.00)	0.00 (0.00–0.00)	0.0046	6.30
SST	Triiodothyronine	ng/mL	0.55–1.92	0.44%	0.97 (0.90–1.02)	0.04 (–0.02–0.11)	0.4259	3.40
SST	Thyroid-stimulating hormone	ng/dL	0.00–7.04	–0.46%	0.99 (0.99–1.00)	0.00 (–0.01–0.01)	0.012	10.00
SST	Free thyroxine	uIU/mL	0.51–2.19	–0.51%	1.01 (0.99–1.07)	–0.02 (–0.08–0.01)	0.1957	2.30
EDTA	White blood cell count	10^9/L	3.89–16.61	1.03%	0.97 (0.93–1.01)	0.32 (0.03–0.55)	0.1279	6.05
EDTA	Red blood cell count	10^12/L	3.25–5.32	–0.89%	1.04 (0.93–1.13)	–0.24 (–0.65–0.28)	0.0374	1.70
EDTA	Hemoglobin	g/dL	10.30–16.70	–0.45%	1.00 (0.95–1.06)	–0.10 (–0.94–0.62)	0.0567	1.84
EDTA	Hematocrit	%	33.10–50.30	0.54%	1.04 (0.92–1.16)	–1.52 (–6.41–3.47)	0.1687	1.70
EDTA	Mean corpuscular volume	fL	77.20–102.80	1.45%	1.03 (0.99–1.07)	–0.94 (–5.20–2.46)	0	1.26
EDTA	Mean corpuscular hemoglobin	pg	24.20–33.50	0.50%	1.00 (0.90–1.11)	0.30 (–3.07–3.24)	0.2007	1.35
EDTA	Mean corpuscular hemoglobin concentration	g/dL	30.80–35.10	–0.97%	1.12 (0.84–1.50)	–4.15 (–16.55–5.03)	0.0041	0.40
EDTA	Red cell distribution width	%	11.40–14.90	–0.22%	1.00 (1.00–1.07)	0.00 (–0.89–0.00)	0.1473	1.70
EDTA	Neutrophil percentage	%	40.70–79.90	–0.23%	0.99 (0.95–1.04)	0.50 (–2.01–3.07)	0.4216	9.25
EDTA	Lymphocyte percentage	%	10.40–44.90	0.83%	1.02 (0.98–1.07)	–0.32 (–1.70–0.92)	0.1194	9.19
EDTA	Monocyte percentage	%	4.20–10.10	–2.67%	1.06 (0.92–1.23)	–0.67 (–1.99–0.42)	0.0485	13.20
EDTA	Eosinophil percentage	%	0.30–6.50	1.57%	1.09 (1.00–1.18)	–0.07 (–0.21–0.10)	0.1116	19.80
EDTA	Basophil percentage	%	0.10–1.40	8.50%	1.00 (0.75–1.00)	0.00 (0.00–0.14)	0.8171	15.40
EDTA	Platelet count	10^9/L	136.00–430.00	–1.17%	1.02 (0.95–1.10)	–9.82 (–32.26–6.65)	0.2007	5.90
EDTA	Mean platelet volume	fL	8.00–11.10	–0.56%	1.00 (0.94–1.08)	–0.10 (–0.87–0.52)	0.0505	2.29
EDTA	Platelet distribution width	fL	7.90–14.20	–0.82%	1.06 (1.00–1.15)	–0.74 (–1.70 to –0.10)	0.1233	1.40
EDTA	Plateletcrit	%	0.14–0.41	–1.90%	1.04 (1.00–1.19)	–0.02 (–0.05 to –0.01)	0.1105	6.00
EDTA	Hemoglobin A1c	%	4.60–8.30	–0.80%	1.00 (0.93–1.00)	0.00 (0.00–0.34)	0.0004	1.40
Sodium citrate	Prothrombin time	sec	11.90–14.40	0.31%	1.00 (0.95–1.21)	0.00 (–2.75–0.61)	0.3724	1.40
Sodium citrate	Activated partial thromboplastin time	sec	28.30–86.10	1.77%	1.08 (0.94–1.22)	–3.00 (–7.46–1.69)	0.0675	1.90
Glucose	Glucose	mg/dL	71.00–368.00	–1.23%	1.00 (1.00–1.02)	–1.00 (–3.87 to –1.00)	0	2.30

Abbreviations: SST, serum separation tube; N/A, not available.

### Stability assessment

When the 24-h results from EDTA tubes were compared with the baseline measurements, mean percentage differences exceeding the allowable bias limits were observed only in BD Vacutainer tubes for MCHC and platelet count, whereas both tube types showed deviations for basophil percentage, mean platelet volume (MPV), PDW, and PCT. Overall, UNI-VAC tubes demonstrated comparable or superior stability at 24 h for most hematological parameters. However, platelet-related indices exhibited higher variability in both tube types, likely reflecting their biological instability (Table 3).

**Table 3:** Stability of hematological parameters at 24 h in Labex UNI-VAC and BD Vacutainer EDTA tubes.

Analyte (unit)	Tube	Baseline median (range)	Mean difference (%)	Allowable bias (%)
White blood cell count (10^9^/L)	Vacutainer	6.12 (3.89–16.61)	–0.39	6.05
White blood cell count (10^9^/L)	UNI-VAC	6.3 (3.89–17.09)	0.63	6.05
Red blood cell count (10^12^/L)	Vacutainer	4.73 (3.25–5.32)	0.52	1.7
Red blood cell count (10^12^/L)	UNI-VAC	4.7 (3.13–5.32)	0.02	1.7
Hemoglobin (g/dL)	Vacutainer	14 (10.3–16.7)	0.39	1.84
Hemoglobin (g/dL)	UNI-VAC	14 (10–16.5)	0.36	1.84
Hematocrit (%)	Vacutainer	42.25 (33.1–50.3)	1.11	1.7
Hematocrit (%)	UNI-VAC	42.45 (32.1–49.3)	0.3	1.7
Mean corpuscular volume (fL)	Vacutainer	91.3 (77.2–102.8)	0.59	1.26
Mean corpuscular volume (fL)	UNI-VAC	92.15 (79–105.4)	0.3	1.26
Mean corpuscular hemoglobin (pg)	Vacutainer	29.95 (24.2–33.5)	–0.1	1.35
Mean corpuscular hemoglobin (pg)	UNI-VAC	30.05 (24.8–34.3)	0.35	1.35
Mean corpuscular hemoglobin concentration (g/dL)	Vacutainer	32.9 (30.8–35.1)	–0.68	0.4
Mean corpuscular hemoglobin concentration (g/dL)	UNI-VAC	32.9 (30.3–34.1)	0.07	0.4
Red cell distribution width (%)	Vacutainer	12.7 (11.4–14.9)	–0.42	1.7
Red cell distribution width (%)	UNI-VAC	12.75 (11.4–14.9)	–0.08	1.7
Neutrophil percentage (%)	Vacutainer	56.75 (40.7–79.9)	–0.13	9.25
Neutrophil percentage (%)	UNI-VAC	57 (40.3–79.9)	0.47	9.25
Lymphocyte percentage (%)	Vacutainer	34.15 (10.4–44.9)	–1	9.19
Lymphocyte percentage (%)	UNI-VAC	33.6 (10.7–44.9)	–1.85	9.19
Monocyte percentage (%)	Vacutainer	7 (4.2–10.1)	4.77	13.2
Monocyte percentage (%)	UNI-VAC	7.2 (4.3–10.3)	5.62	13.2
Eosinophil percentage (%)	Vacutainer	1.6 (0.3–6.5)	7.03	19.8
Eosinophil percentage (%)	UNI-VAC	1.7 (0.2–6.7)	4.95	19.8
Basophil percentage (%)	Vacutainer	0.55 (0.1–1.4)	31.66	15.4
Basophil percentage (%)	UNI-VAC	0.5 (0.2–1.3)	19.7	15.4
Platelet count (10^9/L)	Vacutainer	277 (136–430)	6.16	5.9
Platelet count (10^9/L)	UNI-VAC	277.5 (138–468)	5.39	5.9
Mean platelet volume (fL)	Vacutainer	9.65 (8–11.1)	10.39	2.29
Mean platelet volume (fL)	UNI-VAC	9.75 (8–11)	9.94	2.29
Platelet distribution width (fL)	Vacutainer	10.4 (7.9–14.2)	20.21	1.4
Platelet distribution width (fL)	UNI-VAC	10.25 (7.6–13.7)	18.05	1.4
Plateletcrit (%)	Vacutainer	0.28 (0.14–0.41)	17.34	6
Plateletcrit (%)	UNI-VAC	0.26 (0.14–0.46)	16.1	6
Hemoglobin A1c (%)	Vacutainer	5.5 (4.6–8.3)	–0.32	1.4
Hemoglobin A1c (%)	UNI-VAC	5.5 (4.5–8.2)	0.22	1.4

For sodium fluoride tubes, stability was assessed over 72 h at 4°C. When compared with baseline values, the mean percentage differences at 24, 48, and 72 h were −0.7 ± 1.6%, −1.2 ± 2.2%, and −2.5 ± 2.2% for BD Vacutainer tubes and −1.0 ± 1.5%, −1.4 ± 1.5%, and −2.8 ± 2.4% for UNI-VAC tubes, respectively (Fig 1). Both tubes maintained acceptable stability for up to 48 h based on the predefined allowable bias criteria. However, at 72 h, both tubes exceeded the allowable limit.

**Fig 1:**
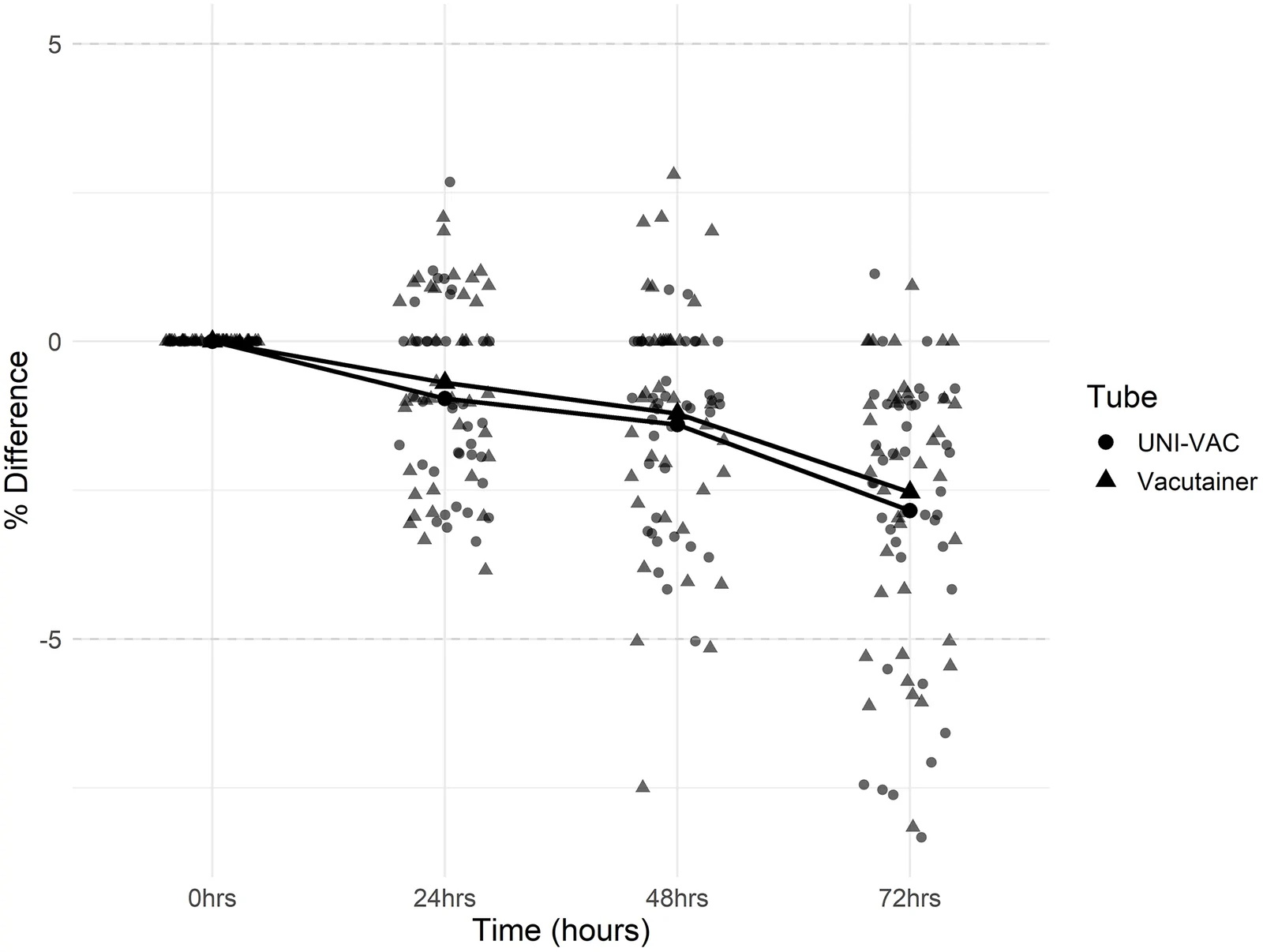
Time-dependent stability of glucose in Labex UNI-VAC and BD Vacutainer sodium fluoride tubes at 4°C.

### Lot-to-lot variability

F-test comparisons showed no significant differences in most hematological indices between BD Vacutainer (two lots) and UNI-VAC (three lots) tubes. The exceptions were platelet count, PDW, and PCT, for which UNI-VAC tubes demonstrated significantly lower standard deviations, suggesting non-inferior lot-to-lot consistency compared with BD Vacutainer tubes (Table 4).

**Table 4:** Lot-to-lot variability of hematological parameters in Labex UNI-VAC and BD Vacutainer EDTA tubes.

Analyte (unit)	Vacutainer		UNI-VAC		F-test	
	SD	CV	SD	CV	F value	P value
White blood cell count (10^9/L)	0.102	1.463	0.106	1.530	0.915	0.611
Red blood cell count (10^12/L)	0.037	0.786	0.033	0.701	1.253	0.213
Hemoglobin (Hb) (g/dL)	0.081	0.575	0.074	0.527	1.182	0.277
Hematocrit (Hct) (%)	0.321	0.760	0.298	0.697	1.157	0.301
Mean corpuscular volume (fL)	0.172	0.190	0.253	0.276	0.461	0.994
Mean corpuscular hemoglobin (pg)	0.219	0.730	0.230	0.765	0.906	0.624
Mean corpuscular hemoglobin concentration (g/dL)	0.237	0.715	0.250	0.762	0.898	0.636
Red cell distribution width (%)	0.047	0.378	0.071	0.564	0.450	0.995
Neutrophil percentage (%)	0.767	1.330	0.795	1.378	0.931	0.589
Lymphocyte percentage (%)	0.580	1.835	0.697	2.212	0.694	0.886
Monocyte percentage (%)	0.424	5.788	0.343	4.623	1.528	0.069
Eosinophil percentage (%)	0.169	6.299	0.192	6.957	0.770	0.805
Basophil percentage (%)	0.119	17.817	0.096	15.957	1.555	0.062
Platelet count (10^9/L)	3.439	1.299	2.683	1.012	1.642	0.042
Mean platelet volume (fL)	0.120	1.192	0.097	0.955	1.554	0.062
Platelet distribution width (fL)	0.492	4.420	0.361	3.163	1.861	0.015
Plateletcrit (%)	0.008	3.008	0.006	2.204	1.800	0.020

## Discussion

In this study, four types of UNI-VAC blood collection tubes manufactured by Labex—serum separator, EDTA, sodium citrate, and sodium fluoride tubes—were compared with BD Vacutainer tubes using 40 clinical specimens. Although paired t-tests revealed statistically significant differences in 20 analytes, all mean percentage differences, except LDH, MCV, and MCHC, remained within the predefined allowable bias limits, indicating that these differences are unlikely to affect clinical decision-making. Our assessment of comparability was therefore based on clinical agreement according to predefined allowable-bias limits rather than on statistical significance alone, since a statistically significant paired t-test does not by itself necessarily denote a clinically important difference. Consistent with this approach, Passing–Bablok regression results were interpreted with emphasis on the magnitude and clinical relevance of any observed bias rather than strict adherence to an intercept of zero. In this context, some analytes showed intercept confidence intervals that did not include zero, indicating the presence of a small constant bias between the two systems; however, these deviations remained within clinically acceptable allowable-bias limits.

LDH is particularly susceptible to hemolytic interference [10], but it is also more sensitive to processing delays, with prolonged serum–cell contact reported to increase LDH levels [11,12]. In this study, LDH values measured with UNI-VAC tubes were approximately 4% lower than those measured with BD Vacutainer tubes. This might be consistent with adequate serum separation in UNI-VAC tubes; however, because hemolysis was assessed only by visual inspection and not quantified using a hemolysis index, this interpretation remains tentative and requires further investigation. Importantly, even for LDH, the observed difference remained below the desirable bias threshold from Westgard and the minimum allowable bias reported in the EFLM Biological Variation Database, indicating that the deviation is unlikely to have clinical significance. MCV and MCHC also marginally exceeded their desirable allowable-bias limits; however, these specifications are extremely narrow (1.26% and 0.40%, respectively) owing to the low biological variation of these indices, and the absolute differences were correspondingly small and unlikely to affect clinical interpretation. Therefore, the results from the UNI-VAC tube can be considered clinically equivalent to those from BD Vacutainer tubes for routine laboratory use.

No visual abnormalities, such as fibrin residues or gel instability, were observed in either tube during centrifugation. However, previous studies have reported issues with BD SST tubes, including the need to remove gel globules or fibrin after centrifugation in 7.5% of cases [13]. A global survey identified gel-related issues in approximately 1% of laboratories, resulting in workflow disruption [14]. Given the relatively small sample size, the absence of such events may reflect the limited number of observations, and the manufacturer’s claims of enhanced gel stability could not be directly confirmed. Large-scale studies are warranted to validate the long-term gel integrity.

For hematology testing, EDTA tubes demonstrated comparable stability to BD Vacutainer tubes after storage at 4°C for 24 h. The 24-h stability assessment was based on prior evidence that most blood count parameters remained stable under these conditions [15]. In this study, platelet-related indices (MPV, PDW, and PCT) showed reduced stability in both tubes, consistent with previous reports identifying instability in MCV, MPV, PDW, and PCT [16,17] and findings that only red blood cell count, platelet count, hemoglobin, and mean corpuscular hemoglobin remained reliable after 24 h at room temperature [18]. Thus, hematology results from EDTA tubes should ideally not be reported after prolonged storage; if unavoidable, cautionary notes should be included for platelet-related parameters. Lot-to-lot variability, assessed by comparing BD Vacutainer (two lots) and UNI-VAC (three lots) EDTA tubes using F-tests, showed no significant differences in standard deviations. Taken together, the EDTA tubes from both manufacturers provide acceptable stability within 24 h with comparable lot-to-lot variability.

The sodium fluoride tube stability was acceptable for up to 48 h for both manufacturers, but a significant decline was observed at 72 h. These results are consistent with those of previous studies, which reported a decrease of approximately 2.5% after 18–26 h of storage [19]. These reductions are attributed to ongoing glycolysis, underscoring the need for either immediate centrifugation (within 10 min) or the use of glycolytic inhibitors when timely processing is not feasible [20]. Accordingly, laboratories should exercise caution when reporting glucose results from delayed analyses.

This study has some limitations. First, the study population did not include a sufficient number of patients with abnormal values. For example, 34 of 40 participants had CRP levels ≤0.3 mg/dL, and none had abnormal sodium levels, limiting evaluation of tube performance across clinically abnormal ranges. Recruiting severely ill patients with electrolyte abnormalities specifically for tube validation was not feasible. Consequently, the equivalence demonstrated here applies primarily to the near-normal concentration ranges sampled; comparability at clinically abnormal or pathological concentrations, particularly for CRP and electrolytes, remains to be established and should not be extrapolated from the present data. In addition, patients with bleeding disorders, severe anemia, or cytopenia were excluded from this study for safety reasons, given the additional research-only venipuncture required by the protocol; tube comparability in these populations was therefore not directly assessed and should not be extrapolated from the present findings. Second, although lot-to-lot variability was assessed, precision testing was not performed in triplicate, as recommended by CLSI GP34-A [9]. Nevertheless, approximate estimates of lot-to-lot variability were obtained by calculating standard deviations across lots. Third, the relatively small sample size may have limited the detection of rare preanalytical issues, such as visible gel instability or fibrin formation. Future large-scale studies incorporating further evaluation of gel integrity under stress and storage conditions are therefore needed to validate the performance of UNI-VAC tubes across broader patient populations. Fourth, the alternating assignment of draw order, rather than formal randomization, may not have fully eliminated order-related bias, although its systematic and balanced nature is expected to have minimized this effect. Lastly, our comparison was based on samples analyzed within one to two hours at room temperature, consistent with the routine practice in our laboratory. In settings where testing is delayed—such as referral laboratories, where samples are transported and analyzed after a delay and therefore require refrigerated (2–8 °C) storage—tube comparability was not evaluated and should be confirmed in future studies.

## Conclusions

In conclusion, the clinical performance of the UNI-VAC serum separator, EDTA, sodium citrate, and sodium fluoride tubes was largely equivalent to that of BD Vacutainer tubes for most analytes. LDH, MCV, and MCHC showed biases marginally exceeding their desirable allowable-bias limits, but the deviations were small and unlikely to be clinically meaningful. The EDTA and sodium fluoride tubes demonstrated comparable stability to BD Vacutainer tubes, with the glucose results being reliable for up to 48 h. The lot-to-lot variability was comparable between manufacturers, although this provides only a preliminary indication of manufacturing consistency, as triplicate precision testing recommended by CLSI GP34-A was not performed. Taken together, these findings suggest that UNI-VAC tubes provide a clinically acceptable alternative to BD Vacutainer tubes in terms of clinical performance and lot-to-lot consistency, without evidence of visible gel instability, although further large-scale validation remains warranted.

## Supporting information

S1 Dataset Raw measurement data. Participant-level paired measurements for all 53 analytes, glucose and EDTA stability, and EDTA lot-to-lot data.
(XLSX)

S1 Fig Bland–Altman plots of analyte agreement between UNI-VAC and BD Vacutainer tubes. Percentage difference (UNI-VAC − BD Vacutainer, relative to BD Vacutainer) is plotted against the mean of paired measurements for each analyte. Lines indicate the bias (blue dashed), 95% limits of agreement (±1.96 SD, red dashed), and the allowable-bias limits (AB; ± %, green solid). C-reactive protein was excluded because most values were below the limit of quantification.
(PDF)
