# Supplementary material for: Evaluation of UNI-VAC serum separator, ethylenediaminetetraacetic acid, sodium citrate, and sodium fluoride tubes: A comparison with Becton, Dickinson and Company Vacutainer tubes
Source: PLoS One. 2026 Oct 5;21(10):e0349489. doi: 10.1371/journal.pone.0349489 (PMC13637866; doi:10.1371/journal.pone.0349489)

S1 Fig. Bland–Altman analysis (page 1 of 6)

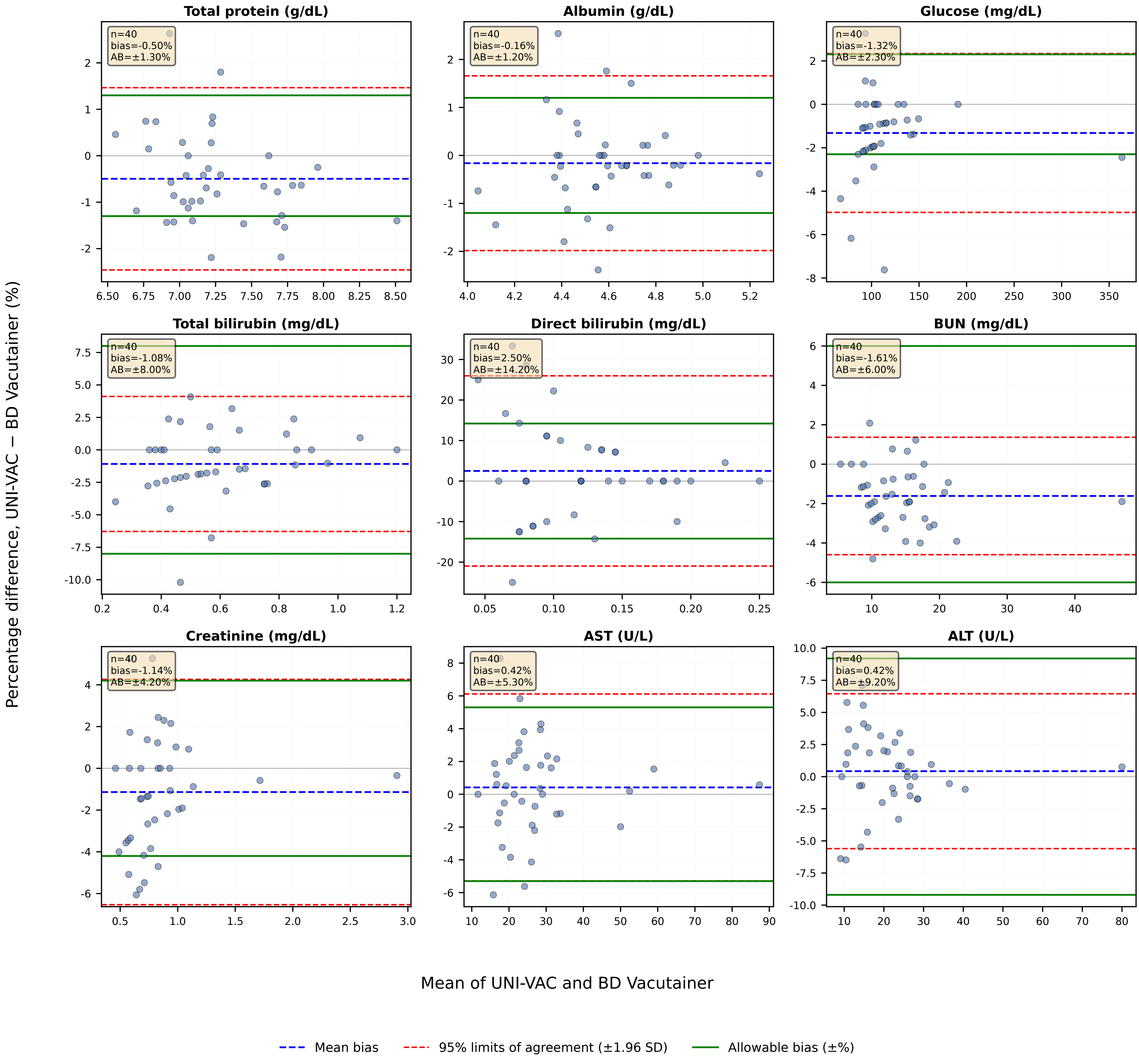

S1 Fig. Bland–Altman analysis (page 2 of 6)

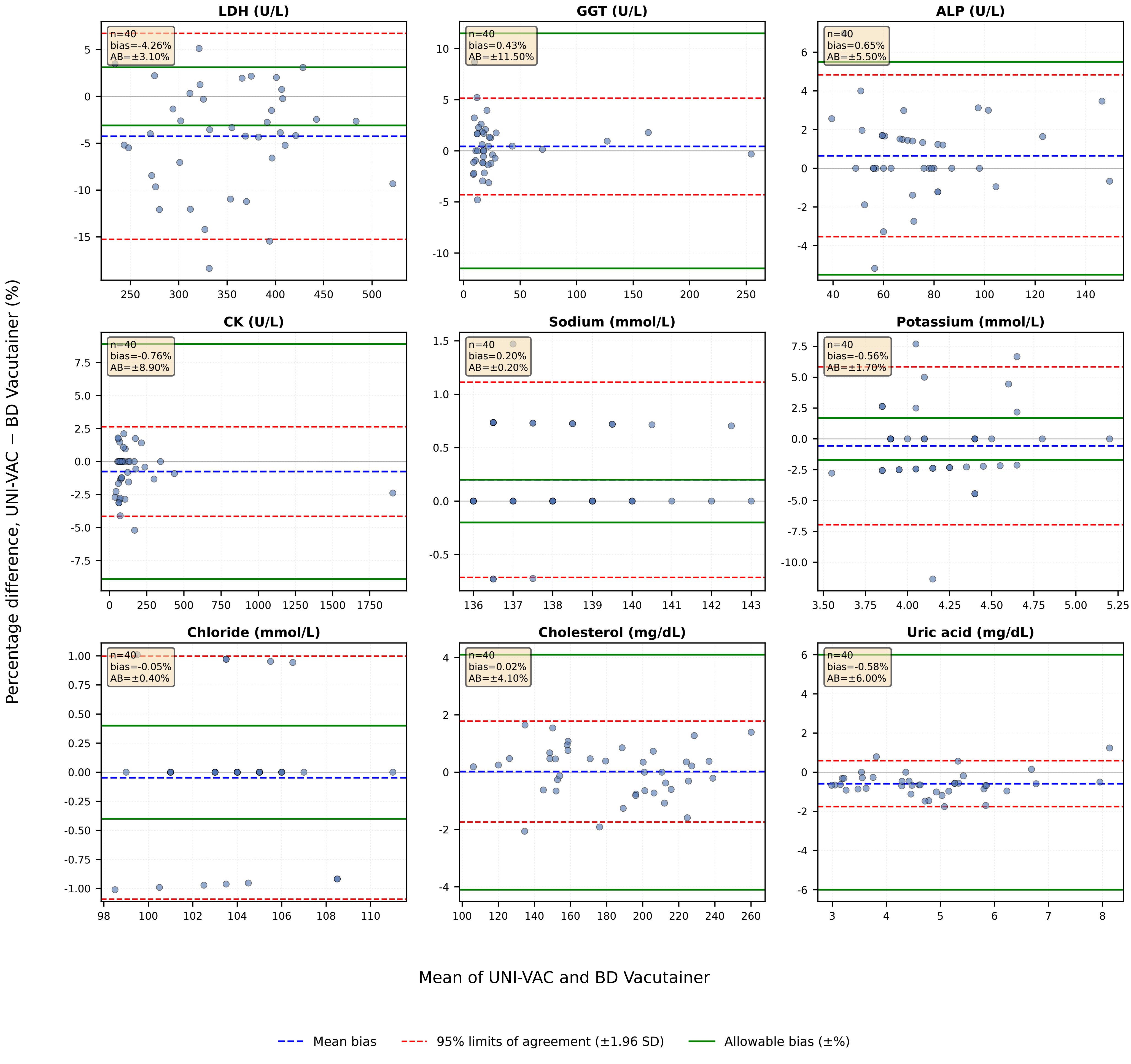

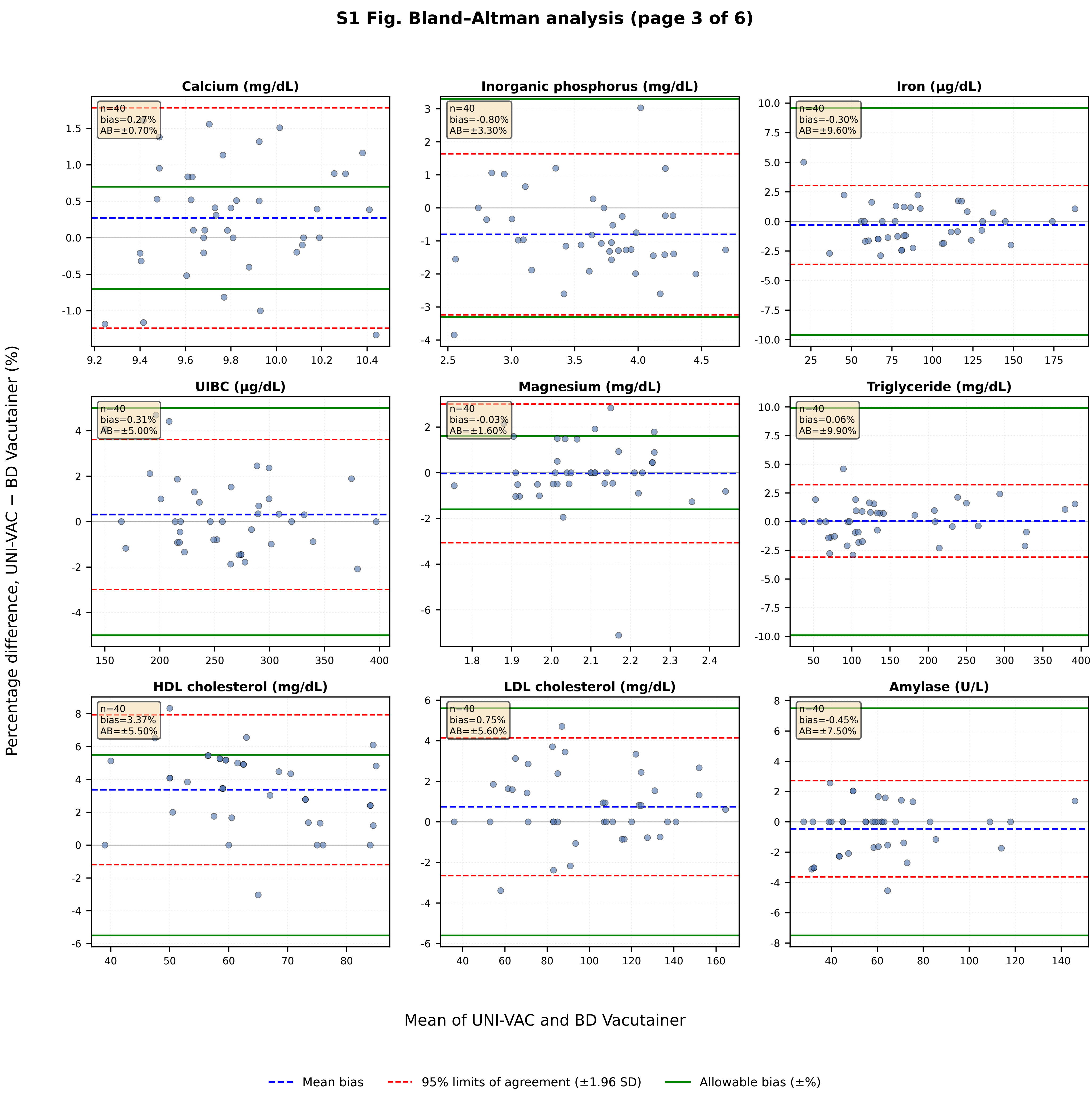
S1 Fig. Bland–Altman analysis (page 3 of 6)
Calcium (mg/dL)
n=40
bias=0.27%
AB=±0.70%
Inorganic phosphorus (mg/dL)
n=40
bias=-0.80%
AB=±3.30%
Iron (µg/dL)
n=40
bias=-0.30%
AB=±9.60%
UIBC (µg/dL)
n=40
bias=0.31%
AB=±5.00%
Magnesium (mg/dL)
n=40
bias=-0.03%
AB=±1.60%
Triglyceride (mg/dL)
n=40
bias=0.06%
AB=±9.90%
HDL cholesterol (mg/dL)
n=40
bias=3.37%
AB=±5.50%
LDL cholesterol (mg/dL)
n=40
bias=0.75%
AB=±5.60%
Amylase (U/L)
n=40
bias=-0.45%
AB=±7.50%
Percentage difference, UNI-VAC − BD Vacutainer (%)
Mean of UNI-VAC and BD Vacutainer
Mean bias
95% limits of agreement (±1.96 SD)
Allowable bias (±%)

S1 Fig. Bland–Altman analysis (page 4 of 6)

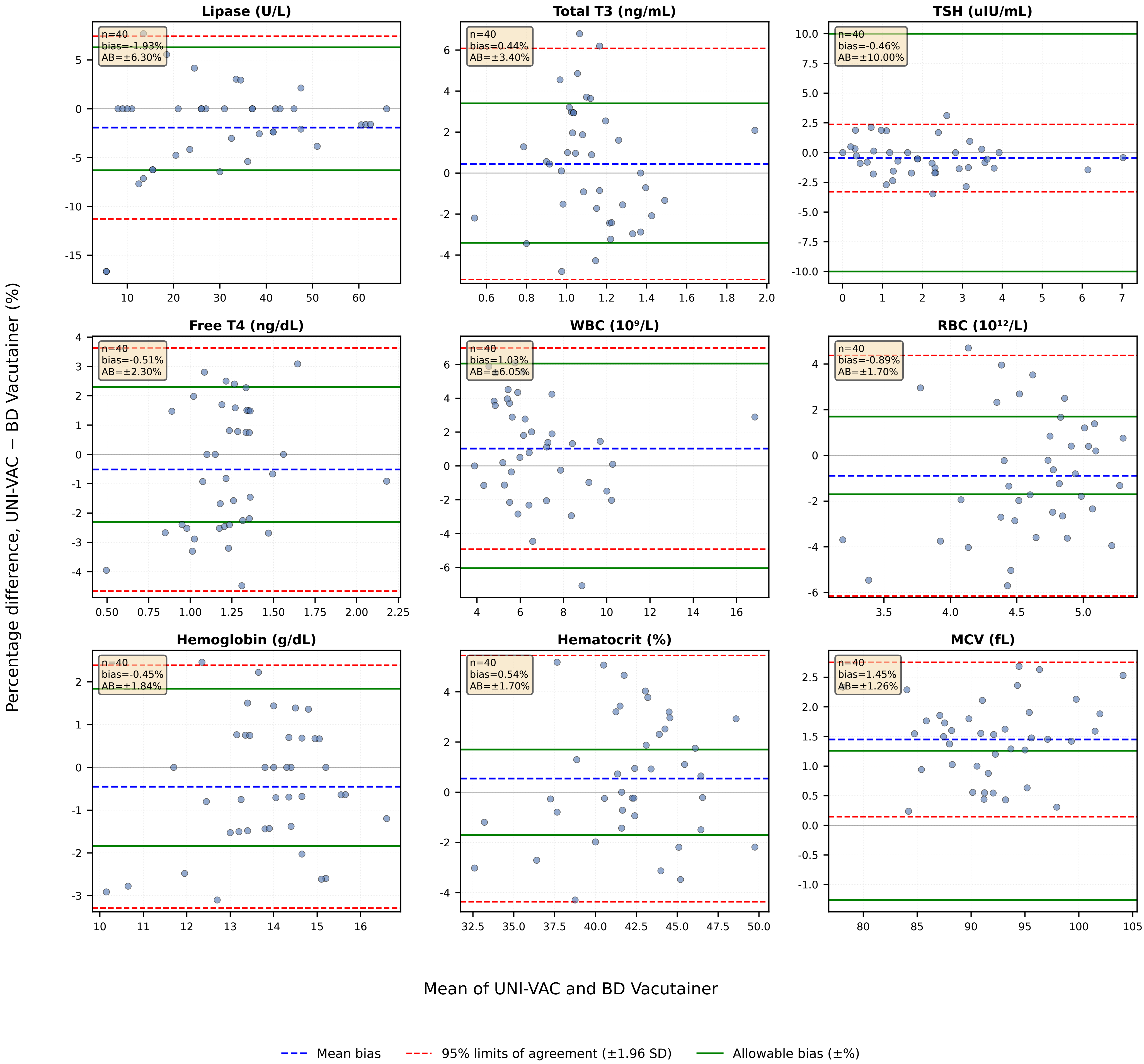

S1 Fig. Bland–Altman analysis (page 5 of 6)

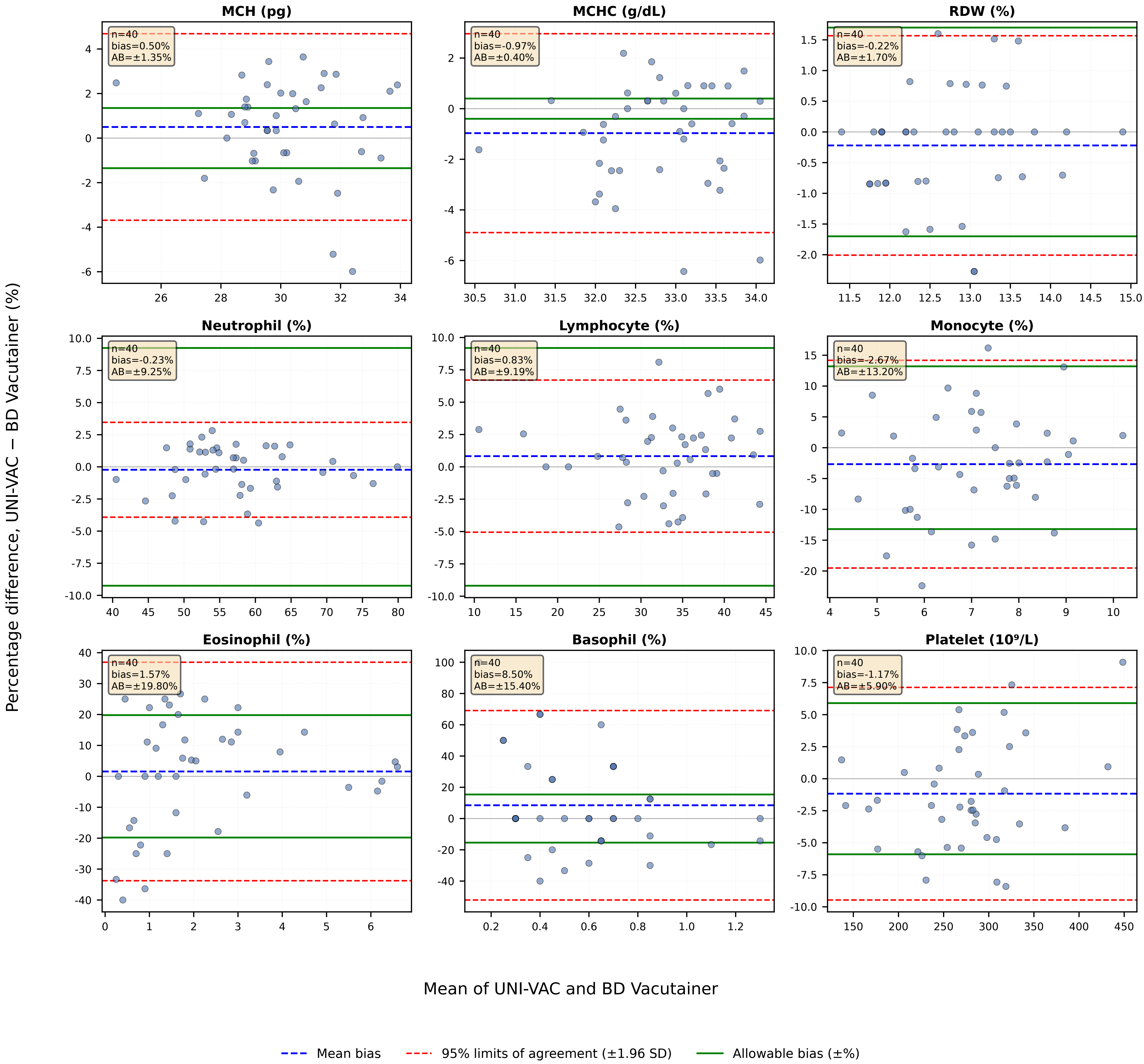

S1 Fig. Bland–Altman analysis (page 6 of 6)

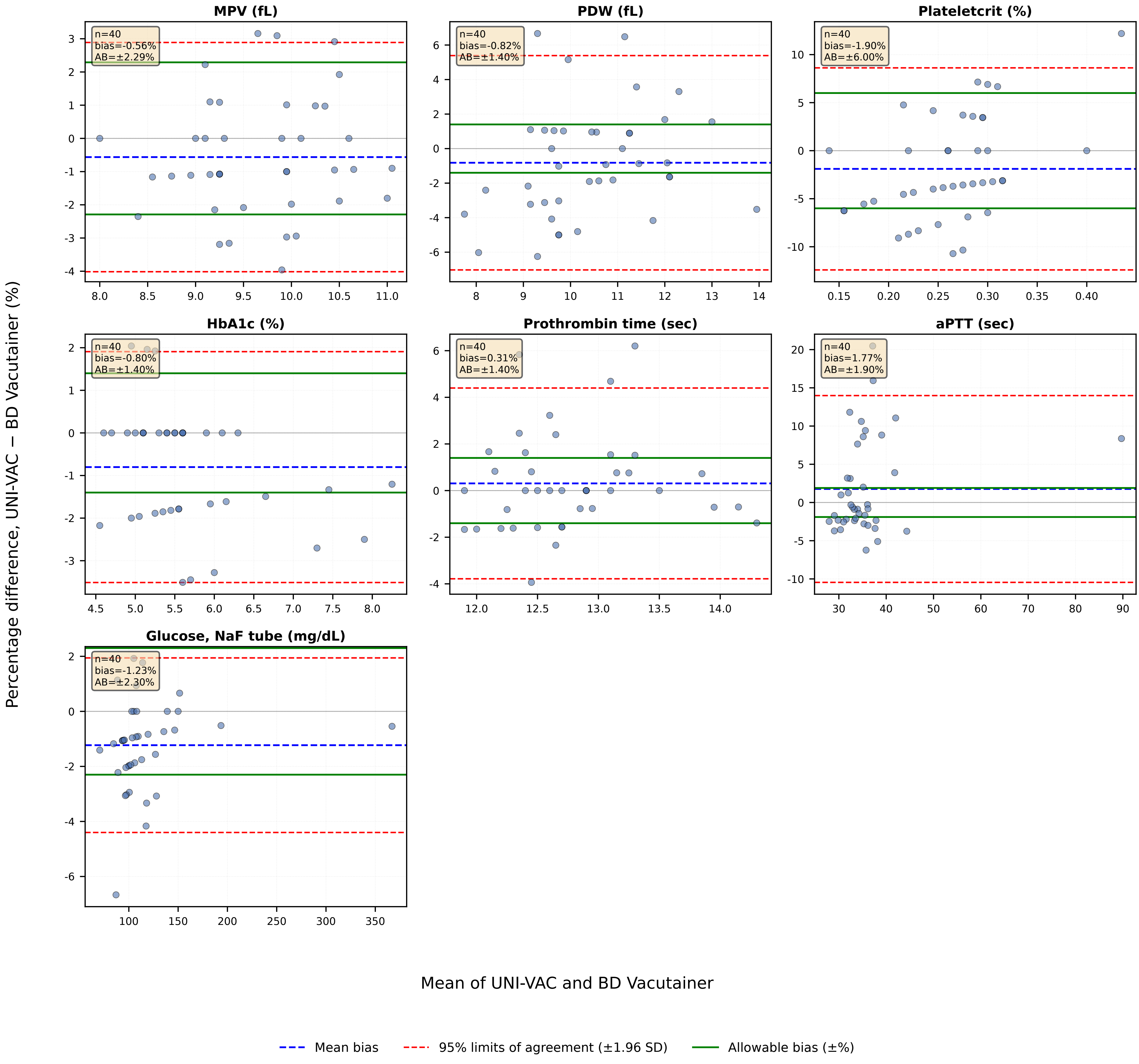

Supplement: Supplementary file 1 [file pone.0349489.s002.pdf]
